# CRMP-2 peptide mediated decrease of high and low voltage-activated calcium channels, attenuation of nociceptor excitability, and anti-nociception in a model of AIDS therapy-induced painful peripheral neuropathy

**DOI:** 10.1186/1744-8069-8-54

**Published:** 2012-07-24

**Authors:** Andrew D Piekarz, Michael R Due, May Khanna, Bo Wang, Matthew S Ripsch, Ruizhong Wang, Samy O Meroueh, Michael R Vasko, Fletcher A White, Rajesh Khanna

**Affiliations:** 1 Department of Pharmacology and Toxicology, 950 West Walnut Street, Indianapolis, IN, 46202, USA; 2 Department of Anesthesia, 950 West Walnut Street, Indianapolis, IN, 46202, USA; 3 Department of Biochemistry and Molecular Biology, Health Information and Translational Sciences Building, 410 W. 10th Street, HS 5000, Indianapolis, IN, 46202, USA; 4 Department of Chemistry, Health Information and Translational Sciences Building, 410 W. 10th Street, HS 5000, Indianapolis, IN, 46202, USA; 5 Program in Medical Neurosciences, Paul and Carole Stark Neurosciences Research Institute, 950 West Walnut Street, Indianapolis, IN, 46202, USA; 6 Department of Center for Computational Biology and Bioinformatics, Indiana University School of Medicine, Health Information and Translational Sciences Building, 410 W. 10th Street, HS 5000, Indianapolis, IN, 46202, USA; 7 Sophia Therapeutics LLC, 351 West 10th Street, Indianapolis, IN, 46202, USA

**Keywords:** Peptide, Excitability, Nociception, AIDS therapy-induced chronic pain, Calcium channels, Molecular dynamics

## Abstract

**Background:**

The ubiquity of protein-protein interactions in biological signaling offers ample opportunities for therapeutic intervention. We previously identified a peptide, designated CBD3, that suppressed inflammatory and neuropathic behavioral hypersensitivity in rodents by inhibiting the ability of collapsin response mediator protein 2 (CRMP-2) to bind to N-type voltage-activated calcium channels (CaV2.2) [Brittain *et al*. Nature Medicine 17:822–829 (2011)].

**Results and discussion:**

Here, we utilized SPOTScan analysis to identify an optimized variation of the CBD3 peptide (CBD3A6K) that bound with greater affinity to Ca^2+^ channels. Molecular dynamics simulations demonstrated that the CBD3A6K peptide was more stable and less prone to the unfolding observed with the parent CBD3 peptide. This mutant peptide, conjugated to the cell penetrating motif of the HIV transduction domain protein TAT, exhibited greater anti-nociception in a rodent model of AIDS therapy-induced peripheral neuropathy when compared to the parent TAT-CBD3 peptide. Remarkably, intraperitoneal administration of TAT-CBD3A6K produced none of the minor side effects (i.e. tail kinking, body contortion) observed with the parent peptide. Interestingly, excitability of dissociated small diameter sensory neurons isolated from rats was also reduced by TAT-CBD3A6K peptide suggesting that suppression of excitability may be due to inhibition of T- and R-type Ca^2+^ channels. TAT-CBD3A6K had no effect on depolarization-evoked calcitonin gene related peptide (CGRP) release compared to vehicle control.

**Conclusions:**

Collectively, these results establish TAT-CBD3A6K as a peptide therapeutic with greater efficacy in an AIDS therapy-induced model of peripheral neuropathy than its parent peptide, TAT-CBD3. Structural modifications of the CBD3 scaffold peptide may result in peptides with selectivity against a particular subset of voltage-gated calcium channels resulting in a multipharmacology of action on the target.

## Background

Neuropathic pain most commonly arises from injury to the nervous system but can also be attributed to disease and/or administration of toxic drugs (i.e. AIDS therapy). Despite a range of available analgesics, neuropathic pain remains a large unmet medical need. The lack of analgesic efficacy may be due to the fact that specific ion channels contribute to the mechanisms underlying the numerous neuropathic pain syndromes. Accumulating evidence identifies adaptive changes in ion channels as a mitigating factor in the molecular etiology of numerous neuropathic pain syndromes.

A variety of voltage-dependent and independent ion channels in addition to nonselective cation channels can influence neurons associated with the nociceptive pathway. Voltage-activated ionic currents are critical to the excitability of nociceptive neurons present in the dorsal root ganglia. Notably, sodium and calcium currents serve to respectively set the threshold and upstroke of action potential (AP) firing as well as facilitating frequency and sustainability of AP firing [1]. While it is generally accepted that an increase in Na^+^ channel density following nerve injury can influence the hyperexcitability of sensory neurons, Ca^2+^ channel subtypes modulate nociceptive signal transduction by influencing synaptic transmission and can enhance cellular excitability by producing subthreshold membrane oscillations and spike firing within the sensory neurons [2]. Voltage-activated Ca^2+^ channels can be classified as sustained high voltage-activated calcium channels (HVACC) and transient low voltage-activated calcium channels (LVACC), also known as T-type (CaV3.1–3). HVACC include N-type (blocked by ω-conotoxin GVIA; CaV2.2), P/Q-type (blocked by ω-agatoxin IVA; CaV2.1) and R-type (resistant to other toxins; CaV2.3). Investigation into the manner in which the LVACC subtypes contribute to depolarizing potential waves and to burst firing following membrane hyperpolarization has yielded evidence to suggest that inhibition of T-type Ca^2+^ currents using the anti-hypertensive drug, mibefradil, can reverse stimulus-dependent neuropathic pain behavior [3]. As mibefradil does not cross the blood–brain barrier, this antihyperalgesic effect is thought to be largely localized to the primary afferent neuron [4-6]. Taken together, these studies position T-type calcium channels as promising therapeutic targets.

In addition to T-type Ca^2+^channels, HVACC R-type Ca^2+^channels have recently been shown to play a role in nociception and neuropathic pain [7,8]. R-type calcium currents are present in rat DRG neurons and have been shown to contribute to neuronal excitability [9-11] as R-type channel knockout mice exhibit reduced responses to inflammatory pain stimuli [7]. In addition, an antagonist of R-type Ca^2+^current, has been shown to contribute to neuronal responses in a spinal nerve ligation (SNL) model of chronic neuropathic pain [8]. While a complete understanding of the role of R-type calcium channels in chronic pain states is in its infancy, these studies suggest the R-type Ca^2+^ channel may be a potential target for analgesics to reduce nociceptive signaling.

Many pharmacotherapeutic agents targeting T- and R-type calcium channels are designed to impact state-dependent transitions. Here, we use an alternative approach: to target modulators of channel trafficking. A recently discovered interaction between the N-type calcium channel, CaV2.2 and collapsin response mediator protein 2 (CRMP-2) positively regulated channel functions by increasing CaV2.2’s cell surface trafficking [12-14]. This CaV2.2 trafficking at the membrane can be effectively disrupted by a short peptide (CBD3) corresponding to a 15 amino acid region of CRMP-2 [12]. CBD3 peptide reduced CaV2.2-mediated currents important for neuropeptide release from sensory neurons and excitatory synaptic transmission in dorsal horn neurons of the spinal cord. Additionally, CBD3 administration *in vivo* reduced hypersensitive behavior in a number of pain models, including a model of neuropathic pain induced by anti-retroviral drug treatment. Here, we tried to improve the parent CBD3 peptide’s efficacy on attenuating neuropathic pain *in vivo* and identify other possible mechanisms underlying its analgesic effects. We were successful with a derivative peptide, TAT-CBD3A6K, whose administration exhibited greater anti-nociceptive effects in a model of AIDS-induced peripheral neuropathy compared to the parent CBD3 peptide. Interestingly, the excitability of dissociated small diameter sensory neurons isolated from antiretroviral therapy-treated rats was reduced by TAT-CBD3A6K likely via an effect on T- and R-type calcium channels. In summary, TAT-CBD3A6K alleviates neuropathic hypersensitivity by preventing CRMP-2-mediated enhancement of T- and R-type calcium channel function, an approach that may prove useful in managing chronic neuropathic pain.

## Results

### CBD3 peptide and identification of mutant CBD3 peptides with altered binding to Ca^2+^channels

**Figure 1:**
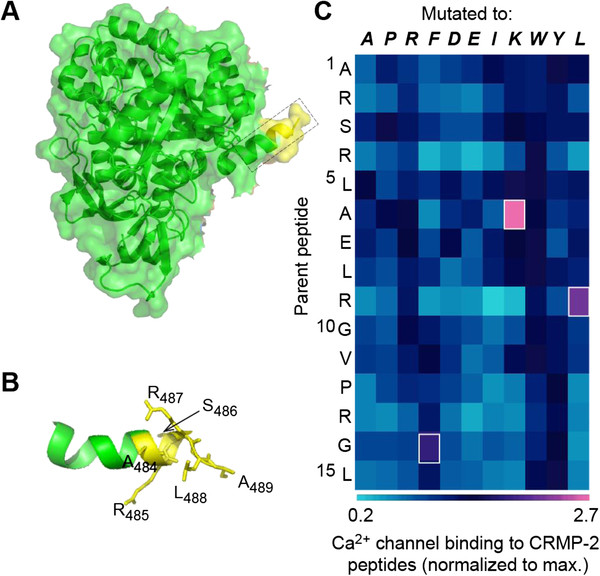
**Scanning mutagenesis of CBD3 identifies better Ca**^**2+**^**channel binding derivative peptides.** (**A**) Superimposed ribbon overlaid on top of surface representations of the three-dimensional structure of the CRMP-2 monomer (RCSB databank PDB code: 2GSE) [79]. The location of the 6 amino acids of the CBD3 peptide are illustrated in the boxed region. (**B**) Ribbon representation of the helix harboring the first 6 amino acids (yellow; denoted in single letter code) of CBD3. (**C**) Matrix representation of normalized binding of CaV2.2 to CRMP-2 peptides singly mutated *in silico* to the indicated amino acids (italicized boldface font). The matrix was generated by Matrix2png [79]. Three peptides with mutations at position 6 (A6K), 9 (R9L) and 14 (G14F) displayed the highest binding to CaV2.2

We had previously mapped several CaV binding domains (CBDs) on CRMP-2 that conferred binding to CaV2.2 [12]. Refinement of the mapping resulted in identification of a 15 amino acid peptide, designated CBD3, which was sufficient to confer the interaction [15]. Fusing CBD3 to the transduction domain of the HIV TAT protein resulted in a cell permeable biologic, which by preventing CRMP-2-mediated enhancement of CaV2.2 function, alleviated inflammatory and neuropathic hypersensitivity [15]. Only 6 of 15 amino acids of CBD3 are present in the CRMP-2 structure (Figure 1A, B) and structure-based homology models derived using a multiple-template threading statistical method (RaptorX, [16]) reveal that the carboxyl 9 amino acids are largely unstructured (data not shown). The lack of structural rigidity and accessibility of CBD3 may be inherently beneficial in blocking protein-protein interactions and offers possibilities for peptide optimization. We hypothesized that single amino-acid mutation scans of the CBD3 sequence may yield superior peptide derivatives not only with respect to CaV2.2 binding but perhaps also with respect to blocking Ca^2+^ channel function, transmitter release, and ultimately hypersensitivity. To this end, we performed a limited mutational scan of the CBD3 peptide and discovered three peptides with point mutations at positions 6 (A6K), 9 (R9L) and 14 (G14F) with greater binding to Ca^2+^ channels than the parent CBD3 peptide (Figure 1C). We have already demonstrated that dural application of TAT-CBD3A6K is better at inhibiting capsaicin (Cap)-evoked meningeal vasodilation in a rodent model of headache pain than the parental CBD3 peptide [17]. This data suggests that peptide optimization strategies do indeed result in sister peptides with enhanced actions, potentially lowering the potential for off-target effects. 

### Molecular dynamics (MD) simulations of wild type and mutant CBD3 peptides

**Figure 2:**
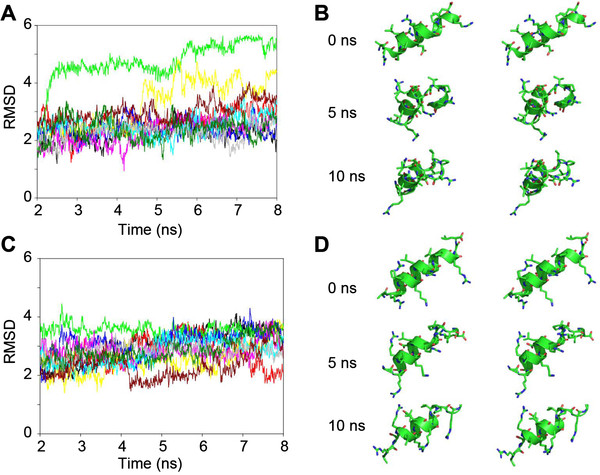
**Molecular dynamics (MD) simulations of wild type and mutant CBD3 peptides.** Conformational change experienced by (**A**) wild type CBD3 and (**C**) CBD3A6K mutant peptides over the course of multiple 10 ns MD simulations. Snapshots at 0, 5 and 10 ns of stereo pairs for (**B**) wild type and (**D**) mutant peptides are shown in capped-sticks representation, color-coded by atom type (C, N, and O in green, blue and red). The overall structure of the peptide is depicted in ribbon representation. RMSD, root mean square deviation (in angstroms)

As a further test of our peptide stability hypothesis, MD simulations were carried out to explore atomistic flexibility of wild type and A6K mutant peptides in solution. A three-dimensional structure for wild type and A6K mutant peptide was constructed. The structure was immersed in a box of explicit-solvent molecules and subjected to 10 separate trajectories consisting of 10 ns of simulation for a total of 100 ns of dynamics per peptide. The evolution of the structure of each peptide over the course of a trajectory is quantified with the root-mean-squared deviation (RMSD), a measure of the deviation of the peptide from its original structure. Following 2 ns of equilibration, the RMSDs for the wild type and CBD3A6K mutant trajectories are shown in Figure 2A and B. For the wild type peptide, the structures of 8 of the 10 trajectories sampled during the course of the simulation fluctuate between 1 and 3 Å. The remaining two wild type trajectories show significant deviation from the initial structure with RMSDs greater than 4 Å, and in one case greater than 5 Å (green curve in Figure 2A). Visualization of this trajectory reveals that at 2 ns, the structure gradually adopts a more compact structure (Figure 2B). In this conformation, the C-terminus amino acid is located near the N-terminus of the peptide (See animation in Additional File 1). The CBD3A6K mutant peptide trajectories show that the RMSD for this peptide also fluctuates between 1 and 4 Å (See animation in Additional File 1). While none of the A6K mutant peptide trajectories showed dramatic conformational changes that were seen in some of the wild type trajectories, mutant structures showed a higher degree of conformational change than wild type. For example, most of the mutant trajectories revealed RMSDs greater than 3 Å starting at 5 ns for the remainder of the simulation. For wild type, only one trajectory showed similar RMSDs. Snapshots for mutant peptide collected at 5 and 10 ns are shown in Figure 2D. Like the wild type peptide, the C-terminus residues are less stable than N-terminus residues, which consistently maintain an α-helical structure throughout the trajectory. Increased conformational change of the mutant peptide may result in greater efficacy for this peptide. Greater conformational change suggests that the peptide may sample alternative conformational states that are more prone to bind to the calcium channel and inhibit its interaction with CRMP-2. The wild type peptide appears more prone to undergo dramatic structural changes that are not seen in the A6K mutant simulations. The lack of such disruptions to the mutant peptide could be another reason that these peptides show greater efficacy.

### TAT-CBD3A6K attenuates AIDS therapy-induced painful peripheral neuropathy

**Figure 3:**
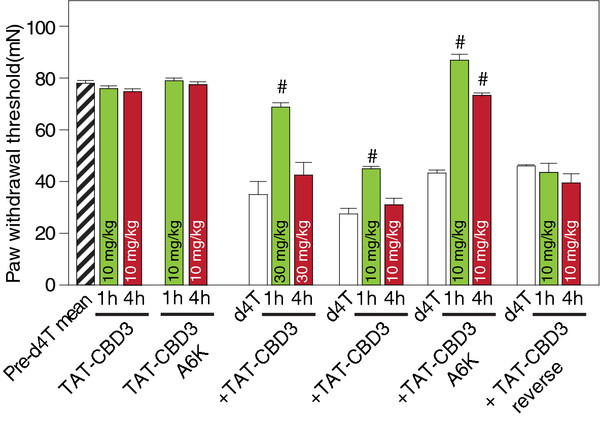
**Effect of TAT-CBD3 and TAT-CBD3A6K peptides on d4T-induced mechanical hypersensitivity.** Paw-withdrawal thresholds were measured in d4T-treated rodents exhibiting mechanical hypersensitivity before and after TAT-CBD3 mutant peptide. Nociceptive thresholds were significantly reduced in d4T-treated rodents (white bars). A single intraperitoneal (i.p.) administration of TAT-CBD3 at 30 mg/kg body weight, but not 10 mg/kg body weight, attenuates d4T-induced mechanical hypersensitivity for at least 1 hour (#, p < 0.01, RMANOVA with Newman-Keul post hoc test; n = 6). Administration of a 10 mg/kg i.p. dose of TAT-CBD3A6K significantly attenuated d4T-induced peripheral hypersensitivity for at least 4 hours (#, p < 0.01, RMANOVA with Newman-Keul post hoc test; n = 6). Administration of a 10 mg/kg i.p. dose of TAT-CBD3 or TAT-CBD3 reverse peptides did not alter d4T-mechanical hypersensitivity (n = 3)

Having identified potentially superior peptides with respect to channel binding, we next tested the efficacy of the best (i.e. A6K) peptide on chronic nociceptive behavior in an animal model of AIDS therapy-induced painful neuropathy [18]. Nucleoside reverse transcriptase inhibitors (NRTIs), part of the highly active antiretroviral treatment (HAART) for AIDS includes drugs such as zidovudine (AZT), zalcitabine (ddC), didanosine (ddI) and stavudine (d4T) that produce side-effects including painful neuropathies. Many of these drugs, including d4T, produce long lasting pain hypersensitivity in humans [19,20] and rodents, but no changes in motor coordination [18]. To study changes in tactile pain sensitivity following a single injection of d4T, we investigated alterations in the threshold force of indentation (produced by von Frey filaments) necessary for eliciting a flexion hindpaw withdrawal reflex. A single administration of d4T (50 mg/kg) produced a significant bilateral decrease in paw withdrawal threshold to von Frey hair stimulation from post-injection day (PID) 3 through the last day of testing at PID14 (p < 0.05; Student’s *t*-test) (Figure 3). 

To determine if TAT-CBD3 and TAT-CBD3A6K could attenuate tactile hyperalgesia elicited by d4T, we administered a single systemic dose of TAT-conjugated versions of CBD3 (30 mg/kg and 10 mg/kg) or CBD3A6K (10 mg/kg) i.p. to d4T-treated rats. Tactile hyperalgesia was assessed 1 and 4 hours following injection of peptides. Systemic injection of either TAT-CBD3 (10 mg/kg) or TAT-CBD3A6K in naïve rats did not alter baseline paw withdraw threshold (PWT) at 1 or 4 hours post injection (pre-d4T mean = 78.0 ± 10.8 mN for control vs. 78.6 ± 1.0 mN 1 hour post TAT-CBD3 and 75.3 ± 0.9 mN 4 hours post TAT-CBD3; p > 0.05, Figure 3) and pre-d4T mean = 74.3 ± 1.0 mN for control vs. 75.7 ± 1.2 mN 1 hour post TAT-CBD3A6K and 74.7 ± 1.2 mN 4 hours post TAT-CBD3A6K; p > 0.05, Figure 3). However, 1 h after 30 mg/kg of TAT-CBD3 was administered to d4T-treated rats; PWT was partially reversed to control levels (68.8 ± 1.7 mN; p < 0.05, Figure 3). The duration of TAT-CBD3’s effect was short lived as PWTs returned to pre-peptide levels by 4 hours (42.5 ± 4.9 mN). A lower dose of TAT-CBD3 (10 mg/kg) also significantly attenuated d4T-elicited hyperalgesia albeit to a lesser degree than the 30 mg/kg dose. One h after 10 mg/kg of TAT-CBD3 was administered to d4T rats, PWT was higher by ~64% compared to pre-peptide levels (p < 0.05, Figure 3). Similar to the 30 mg/kg dose, PWTs returned to predosing levels 4 h post 10 mg/kg TAT-CBD3 administration (31.0 ± 2.5 mN). Remarkably, a 10 mg/kg dose of the derivative peptide TAT-CBD3A6K had greater efficacy in attenuating d4T-elicited hyperalgesia than that of the parent TAT-CBD3 at a dose of 30 mg/kg. 1 h after 10 mg/kg of TAT-CBD3A6K was administered to d4T rats, PWT was fully reversed to control levels (82.2 ± 0.8 mN; p > 0.05; repeated measures analyses of variance (RMANOVA) followed by post hoc pairwise comparisons (Student-Newman-Keuls Method) and significantly higher pre-peptide levels (43.3 ± 1.10 mN; p < 0.05 Figure 3). Additionally, PWTs were significantly higher than pre-peptide dosing levels by 4 hours (72.9 ± 1.3 mN, p < 0.05, Figure 3). All effects of TAT-CBD3 peptides returned to pre-peptide levels 24 h post injection (data not shown). A reverse of TAT-CBD3 peptide (TAT-CBD3 reverse) had no effects on PWT at 1 and 4 hours post injection (p > 0.05, Figure 3). These results suggest that the TAT-CBD3 and TAT-CBD3A6K peptides are effective in blocking AIDS therapy-induced painful peripheral neuropathy.

### TAT-CBD3 peptides reduce the excitability of nociceptive DRG neurons

**Figure 4:**
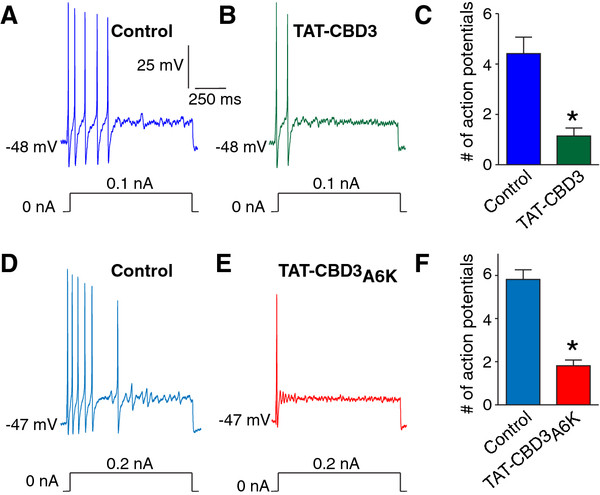
**TAT-CBD3 and TAT-CBD3A6K peptides reduce the excitability of nociceptive dorsal root ganglia (DRG) neurons.** Current clamp recordings were performed on small-to-medium (>30 μm– >40 μm) diameter lumbar 4–5 DRG neurons from d4T-treated rats. Firing of 4–6 action potentials (APs) was elicited by a 1 second depolarizing current injection (ranging from 0.1 to 0.4 nA depending on the cell) every 30 seconds (**A, D**). Representative recordings demonstrating that application of 10 μM CBD3 and CBD3A6K reduces the number of elicited action potentials (**B, E**). Group data showing that TAT-CBD3 (**C**) and TAT-CBD3A6K (**E**) caused a significant reduction in DRG action potential firing (*, p <0.05 versus control, Student’s *t*-test; n = 8 each)

Peripheral neuropathy is often a result of hyperexcitability of peripheral sensory neurons. Compounds that have been shown to inhibit sensory neuron excitability also attenuate neuropathic pain [21]. Additionally, the attenuation of neuropathic pain with peripherally acting compounds such as mibefradil suggests that its analgesic effects are likely via action on peripheral sensory neurons. As our parent peptide, TAT-CBD3, was shown to attenuate neuropathic pain through a decrease in transmitter release and excitatory synaptic transmission in the spinal cord via a reduction in N-type calcium current [15], it was plausible that this peptide also acts on calcium channels that control neuronal excitability in neurons, namely the T- and R-type calcium channels [22,23]. However, this possibility was not previously explored. Thus, to examine the possibility that these peptides affect the neuronal excitability of dissociated DRG neurons, we tested small-to-medium (>30 μm – >40 μm) sensory neuron excitability before and after addition of 10 μM TAT-CBD3 and TAT-CBD3A6K. We used current clamp recordings to test for possible direct effects of TAT-CBD3 and TAT-CBD3A6K on the overall excitability of DRG neurons from d4T-treated rats. The excitability was measured by injecting current pulses (nA) into the soma of small diameter DRG neurons every 30 seconds in order to elicit 4–6 action potentials (average current injection of 0.6 ± 0.1 nA (n = 16)) under control conditions prior to the addition of peptides into the recording bath. Representative recordings (Figure 4A, B, D, E) and grouped data (Figure 4C, F) show that the excitability of small diameter DRG neurons was decreased by both TAT-CBD3 and TAT-CBD3A6K. Five minutes following perfusion of CBD3, there was on average a ~74.1% reduction in the number of action potentials elicited by a current pulse compared to control (1.1 ± 0.3 APs with TAT-CBD3 versus 4.4 ± 0.7 APs without TAT-CBD3 (n = 8) (Figure 4A, B, C; *, p <0.05, Student’s *t*-test). Additionally, five minutes following perfusion of TAT-CBD3A6K, there was on average a ~68.8% reduction in the number of action potentials elicited by a current pulse compared to control (1.8 ± 0.3 APs with TAT-CBD3A6K versus 5.8 ± 0.4 APs without TAT-CBD3A6K (n = 8) (Figure 4D, E, F; *, p <0.05, Student’s *t*-test). Both TAT-CBD3 and TAT-CBD3A6K peptides failed to alter the neuronal resting membrane potential (control −50.9 ± 1.4 mV (n = 8) vs. –50.4 ± 1.7 mV (n = 8) following CBD3 application; and control −54.1 ± 2.7 mV (n = 7) vs. –55.3 ± 3.1 mV (n = 7) for A6K; p > 0.05 for both TAT-CBD3 and TAT-CBD3A6K). These results suggest that DRG excitability can be affected by TAT-CBD3 peptides. 

### Effect of TAT-CBD3 and TAT-CBD3A6K peptides on T- and R-type calcium currents in DRG neurons

We previously demonstrated that TAT-CBD3 reduces HVACC, most notably N-type calcium currents in DRG neurons [15]. Whether T- and R-type calcium currents were affected by the CBD3 peptide was not explored. Based on the observations that: (i) T-type channels are available for opening only from very negative membrane potentials and are thus ideally positioned for regulating neuronal excitability [2,9,24,25], (ii) R-type channels have been shown to contribute to cellular excitability by affecting afterdepolarization and burst firing of pyramidal hippocampal neurons [23], and (iii) our findings that DRG excitability can be affected by CBD3 peptides, we asked if CBD3 peptides affect T- and R-type calcium currents in DRGs. For these studies, we focused primarily on the TAT-CBD3A6K peptide as MD simulations showed a higher propensity of conformational change which may be responsible for the greater efficacy observed for the peptide at attenuating neuropathic pain, combined with its lack of effects on whole-body contortion or tail kinking observed with the parent TAT-CBD3 peptide (n = 6 each), and equal, if not better efficacy than the parent TAT-CBD3 in suppressing DRG excitability.

**Figure 5:**
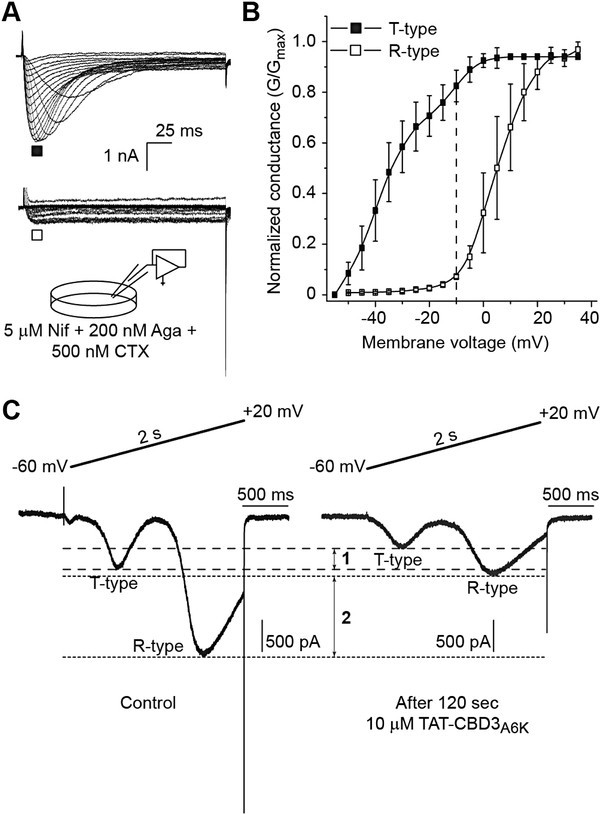
**Pharmacological and biophysical dissection of TAT-CBD3A6K-mediated block of T-and R-type calcium currents in DRG neurons.** (**A**) Representative T- (top) and R-type (bottom) current traces obtained from two separate DRG neurons evoked by 200 ms steps in 5 mV increments from −60 mV to +50 mV, from a holding potential of −90 mV. The extracellular bath solution contained 5 mM Nifedipine (Nif), 200 nM ω-Agatoxin IVA (Aga) and 500 nM ω-Conotoxin GVIA (CTX) to block L-, P/Q-, and N-type calcium currents, respectively. (**B**) Summary of the normalized conductance (G) versus voltage relations for DRG neurons with T- (filled squares) or R- (open squares) type calcium currents. The dotted line at −10 mV highlights the clear discrimination in conductances between T- and R-type currents. (**C**) Representative currents, evoked by a ramp depolarizations from −60 mV to +20 mV for 2 s, illustrating the presence of both T- and R-type currents in the same DRG neuron before (left trace) and 2 min after application 10 μM TAT-CBD3A6K (right trace)

Whole-cell voltage clamp recordings were made from acutely dissociated small-to-medium (>30 μm - >40 μm) diameter sized rat lumbar 4–5 DRG neurons. In order to isolate calcium currents in DRG neurons recordings were acquired with an extracellular solution containing 500 nM TTX and 110 mM N-methyl-d-glucamine to suppress inward sodium currents. Additionally, 5 μM Nifedipine, 500 nM ω-Conotoxin GVIA, and 200 nM ω-Agatoxin IVA were added to the extracellular recording solution to block L-, N- and P/Q-type currents, respectively. Under such recording conditions recorded Ba^2+^ currents can only be carried by the T- or R-type calcium currents. Whole-cell Ba^2+^ currents were elicited from a holding potential of −90 mV to depolarizing test potentials (ranging from–60 mV to +50 mV in 5 mV increments) for 200 ms. Figure 5A shows representative voltage-dependent inward currents (*I*_Ba_) from DRG neurons with fast kinetic properties (i.e. T-type, top traces) similar to those reported previously [2] as well as those with kinetically slow (i.e. R-type, bottom traces) properties. A normalized conductance versus condition voltage plot (Figure 5B) shows clear separation between LVACC (T-type) from HVACC (R-type) currents: at −10 mV, T-type calcium currents contribute to >80% of the conductance with R-type contributing <3% (Figure 5B, dotted line). Using a ramp protocol (depolarizations from −60 mV to +20 mV for 2 sec) which allows for rapid screening of the presence of LVACC and HVACC, we observed both T- and R-type currents in the same DRG (Figure 5C) neuron which were inhibited by ~35% and ~55%, respectively, within 2 minutes of application of TAT-CBD3A6K (Figure 5C). 

**Figure 6:**
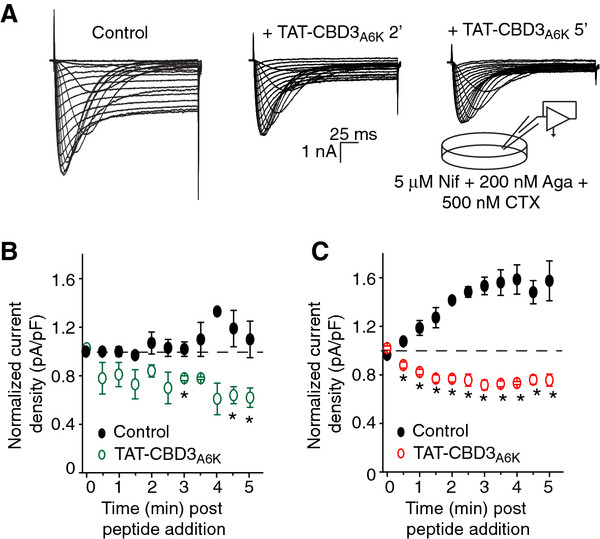
**Characterization of TAT-CBD3A6K-mediated inhibition of T- and R-type calcium currents.** (**A**) Representative family of traces from a DRG neuron with both T- and R-type calcium currents before (*left*), 2 min (*middle*) and 5 min (*right*) after addition of 10 μM TAT-CBD3A6K. Currents were elicited in response to the voltage protocol described in the legend to Figure 5A. To isolate T- and R-type calcium currents, the extracellular bath solution contained 5 mM Nifedipine (Nif), 200 nM ω-Agatoxin IVA (Aga) and 500 nM ω-Conotoxin GVIA (CTX) to block L-, P/Q-, and N-type calcium currents, respectively. (**B**, **C**) Time course of TAT-CBD3A6K mediated inhibition (“run-down”) of T-type (**B**) and R-type (**C**) calcium currents. Time course of inhibition is shown as averaged normalized current density (pA pF^-1^) before peptide addition and at intervals of 30 s for 5 min. Averaged values are shown with standard error for 4–6 control cells and 4 cells following addition of 10 μM TAT-CBD3A6K. The asterisk denotes statistical significance (p < 0.05; Student’s *t*-test) compared to the corresponding control time point. Some error bars are smaller than the symbols. Data represent mean ± SEM from n = 3–6 cells at each time point except for n = 2 the 4 min time point for T-type currents in the presence of peptide

In order to better characterize the extent of the peptide-mediated inhibition of calcium currents, we performed time course experiments with TAT-CBD3A6K. Figure 6A shows a representative family of T- and R-type calcium currents in a DRG before (leftmost traces), at 2 min following bath perfusion of 10 μM TAT-CBD3A6K (middle traces), and at 5 min after the peptide was added (rightmost traces). Representative currents, elicited at −10 mV — a voltage at which R-type currents account for <3% of the current — show a progressive development of peptide-mediated inhibition of T-type current (Figure 6B). Five minutes following perfusion of TAT-CBD3A6K, there was a ~43.6% reduction in peak T-type currents compared to control (normalized current density: 0.62 ± 0.08 (n = 3) with TAT-CBD3A6K versus 1.1 ± 0.1 (n = 3) without TAT-CBD3A6K (Figure 6B; *, p <0.05, Student’s *t*-test). At the same time following perfusion of CBD3A6K, there was a ~52.0% reduction in peak R-type currents compared to control (normalized current density: 0.76 ± 0.05 (n = 3–6) with TAT-CBD3A6K versus 1.6 ± 0.2 (n = 3–6) without TAT-CBD3A6K (Figure 6C; *, p <0.05, Student’s *t*-test). Significant run up of LVACC and HVACC was observed in our recordings, consistent with previous reports [26-28]. Nevertheless, the inhibition occurred faster for the R-type currents compared with the T-type currents but plateaued at around 4 min following addition of peptide. 

**Figure 7:**
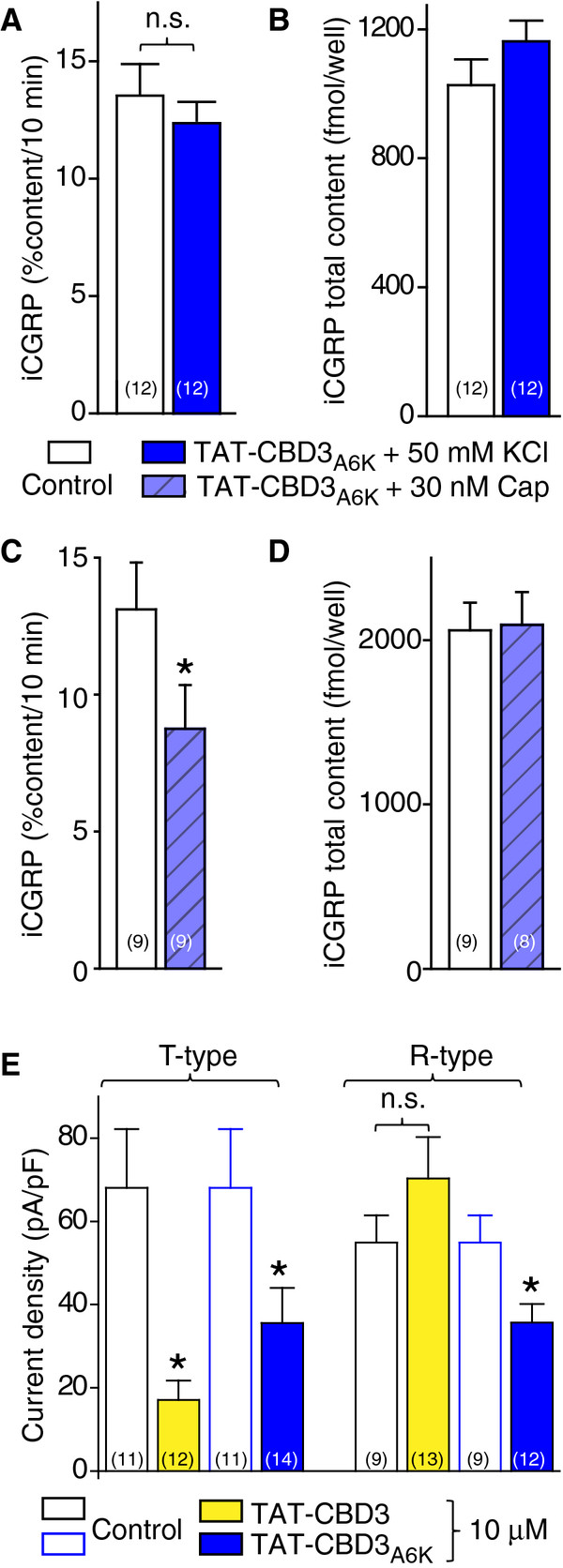
**Effects of TAT-CBD3A6K on evoked transmitter release and TAT-CBD3 and TAT-CBD3A6K on T- and R-type calcium currents.** (**A, C**) Adult rat DRG neurons were maintained in culture for 12–14 days prior to the release experiments. Bar graph of immunoreactive calcitonin gene-related peptide (iCGRP) release expressed as mean percent total iCGRP content of cells in each well ± s.e.m. (n = 9-12 wells/condition as indicated). Neuropeptide release was measured from cells treated with normal HEPES buffer containing 3.5 mM KCl, HEPES buffer containing 10 μM TAT-CBD3A6K, HEPES buffer containing 50 mM KCl or 30 nM Capsaicin (Cap), followed by a final HEPES buffer containing 3.5 mMKCl again. DRGs were exposed to vehicle or TAT-CBD3A6K peptide, at 10 μM, included in the 10 minutes prior to and throughout the high (50 mM) potassium (**A**) or 30 nM capsaicin (**C**) challenges. The resulting total TAT peptide exposure time was for 30 min. There was no significant difference in the high potassium-evoked iCGRP release (**A**) between TAT-CBD3A6K and the control (no treatment) using an ANOVA with Dunnett’s post-hoc test (p > 0.05). The Cap-evoked iCGRP release significantly less in neurons treated with TAT-CBD3A6K compared to control neurons (**C**, *, p < 0.05; ANOVA with Dunnett’s post-hoc test). The evoked release was obtained by subtracting iCGRP release during the two initial basal fractions from that during the potassium or capsaicin-stimulated fractions and expressing it as percent of total iCGRP content in each group. In all cases, release stimulated by high potassium or capsaicin was significantly higher than basal release. (**B, D**) The total content of iCGRP measured at the end of the release experiments for either high potassium (**A**) or 30 nM capsaicin (**C**) challenges. There were no significant differences in iCGRP content between the conditions tested (p > 0.05). (**E**) Bar graphs illustrating the average T- and R-type calcium current densities (pA pF^-1^) ± SEM values for DRGs treated with vehicle (control; open white and open blue bars) or 10 μM TAT-CBD3 (yellow) TAT-CBD3A6K (blue) bath-applied for at least 10 min. Maximal current inhibition for both T- and R-type currents was observed at ~5 min and plateaued thereafter. The number of cells are indicated in parentheses. The asterisk denote statistical significance (p < 0.05; one-way analysis of variance with Dunnett’s post-hoc test) compared to the untreated control cells

Since development of current inhibition develops slowly and stable recordings (lasting ≥10 min) with peptide were not readily obtainable, we chose to further examine peptide-mediated inhibition of T- and R-type currents in bath-applied population studies. These measurements were conducted at least 10 min following addition of peptides, a time point well beyond that at which all run up phenomena has normalized. As selective blockers or R- and T-type currents were not used in these studies, cells with only R-type or only T-type currents were used in the analyses with T-type being measured at −10 mV and R-type at +20 mV; at the latter voltage almost all T-type current is inactivated [29]. T-type calcium current density was decreased by ~75% and ~48% by TAT-CBD3 and TAT-CBD3A6K, respectively (V_t_ = −10 mV, p <0.05, one-way analysis of variance versus respective controls; see Figure 7E). Additionally, the R-type calcium current density was decreased by ~35% (V_t_ = +20 mV, p <0.05, one-way analysis of variance versus control) in TAT-CBD3A6K-treated neurons (see Figure 7E). In contrast, TAT-CBD3 did not affect R-type currents: 54.9 ± 6.5 (n = 9) in control DRGs versus 70.3 ± 9.9 (n = 13) pA pF^-1^ in TAT-CBD3-treated neurons. In summary, TAT-CBD3A6K peptide blocks both T- and R-type calcium currents in sensory neurons. 

### Effect of TAT-CBD3A6K on evoked transmitter release from isolated sensory neurons

We previously demonstrated that TAT-CBD3 reduces release of the neuropeptide transmitter calcitonin gene related peptide (CGRP) from sensory neurons [13]. Here we asked if TAT-CBD3A6K could have a similar effect. Two stimulation paradigms were used to evoke release from all (with 50 mM potassium chloride; KCl) or presumptive nociceptive (with 30 nM Capsaicin; Cap) DRGs. The KCl- or Cap-stimulated release of CGRP was measured from sensory neurons exposed to vehicle or TAT-CBD3A6K (10 μM) included in the 10 minutes prior to and throughout the high K^+^/Cap exposures (total peptide exposure of 30 min). The levels of basal or resting release of immunoreactive CGRP (iCGRP) were not significantly different between any of the groups tested: (for high K^+^ experiments: 1.67 ± 0.14% total peptide content/10 min (n = 12 wells) in untreated control neurons compared to 1.32 ± 0.10% total peptide content/10 min (n = 12 wells) in TAT-CBD3A6K peptide-treated neurons; for Cap-stimulated experiments: 1.73 ± 0.25% total peptide content/10 min (n = 9 wells) in untreated control neurons compared to 1.30 ± 0.17% total peptide content/10 min (n = 9 wells) in TAT-CBD3A6K-treated neurons). A 10 min stimulation with 50 mMKCl or 30 nM Cap evoked a robust increase (~10-fold over basal) in iCGRP release in untreated and TAT-CBD3A6K-treated neurons (Figure 7A, C, p > 0.05, ANOVA). The evoked release, obtained by subtracting iCGRP release during the two initial basal fractions from that during the potassium or capsaicin-stimulated fractions and expressing it as percent of total iCGRP content in each group, was not different between untreated and TAT-CBD3A6K-treated neurons in experiments with K^+^ evoked release (Figure 7A, p > 0.05). The total cellular content of iCGRP was also not different between untreated and TAT-CBD3A6K-treated neurons (Figure 7B, p > 0.05). In contrast, in experiments with Cap, the evoked release was ~32% less in neurons treated with TAT-CBD3A6K compared to control neurons (Figure 7C, p < 0.05; ANOVA with Dunnett’s post-hoc test). The decrease in Cap-stimulated iCGRP release observed in TAT-CBD3A6K-treated neurons was not caused by an increase in the total cellular content of iCGRP as there was no significant difference in neuropeptide content between the two conditions (Figure 7D). These results show a stimulus-dependent effect of TAT-CBD3A6K on iCGRP release from sensory neurons.

## Discussion

**Figure 8:**
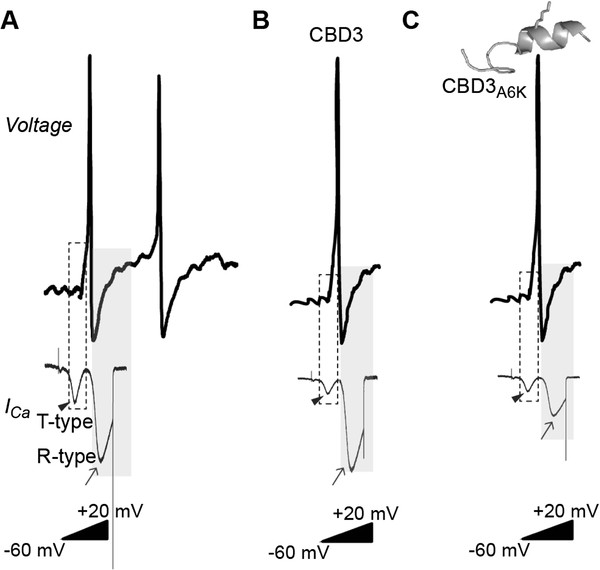
**Schematic summarizing effects of CBD3A6K peptide on excitability and T- and R-type calcium channels.** The figure shows a tentative model of the effects of CBD3A6K on an action potential recorded from a primary afferent neuron exhibiting T-type LVACC (*arrowheads*) and R-type (CaV2.3) HVACC (*arrows*). (**A**) The action potential was elicited by the injection of a depolarizing current. CBD3 blocks the T- but not R-type currents (**B**) while CBD3A6K has defined blocking effects on both the T- and the R-type currents (**C**). T-type calcium currents generally make little contribution to the rising phase of action potentials because their activation kinetics are slower than sodium channels. However, R-type calcium channels typically begin to be activated near the peak of the action potential and this calcium current is largest during the falling phase, when channels have been opened and the driving force on calcium increases. Whether the rapid calcium entry via R-type channels contributes to the activation of large conductance calcium-activated potassium channels is unknown

Neuropathic pain can result from primary dysfunction of peripheral nociceptive sensory neurons. This pain outlasts the early stage of injury and frequently leads to debilitating disorders that are not responsive to conventional analgesic drug therapies. It has been suggested by a number of groups that regulation of voltage-activated calcium channels may contribute to hyperexcitability of the primary afferent neuron associated with chronic pain states [21,30-33]. An emerging approach to treating neuropathic pain is to target ion channels on sensory neurons that play a role in neuronal excitability and neurotransmitter release. Two candidate ion channels have been shown to control neuronal excitability and neurotransmitter release in peripheral sensory neurons: the T- and N-type calcium channels, respectively. Additionally, R-type calcium channels can control neuronal burst firing. As was previously reported, a peptide of CRMP-2 fused to the HIV transactivator of transcription (TAT) protein (TAT-CBD3) decreased neuropeptide release from sensory neurons and excitatory synaptic transmission in dorsal horn neurons via inhibition of CaV2.2 [15]. This peptide also significantly reduced nocifensive behavior induced by formalin injection or corneal capsaicin application and reversed neuropathic pain produced by an antiretroviral drug [15]. Here we report that a TAT-conjugated peptide derivative of CBD3, TAT-CBD3A6K, may decrease neuronal excitability of DRG sensory neurons via inhibition of T- and R-type calcium currents and attenuates neuropathic pain in the Stavudine (d4T)/Zerit®) model of AIDS-therapy induced peripheral neuropathy at concentrations substantially lower than required for the parent TAT-CBD3 peptide. Our data suggests that the TAT-CBD3A6K peptide affects neuropathic pain signaling by blocking T-, R-, and/or N-type calcium channels on primary sensory neurons. That the T- and R-type calcium channels are thought to play an important role in the genesis of repetitive firing and pace-making activity in neurons [34] suggests that these calcium channels have a potentially unique function in primary afferent neuronal excitability (Figure 8) [34-37]. 

The precise physiological function of DRG neurons expressing T- and R-type Ca^2+^ channels is not fully understood. The involvement of R-type channel in modulation of hyperexcitability of DRG neurons has yet to be discerned, but given their function in controlling burst firing in other neurons in the CNS, they may prove to have similar function in the PNS. In contrast, T-type Ca^2+^ channels in DRG have been shown to be involved in modulating the excitability of DRG neurons [37,38]. Low voltage-activated calcium channels (T-type) are present in small-, medium- and large-sized DRG neurons [37]. Three subtypes of T-type Ca^2+^ channels exist (CaV3.1–3), however in the peripheral nervous system, CaV3.2 is the predominant subtype in the rat DRG [39]. CaV3.2 has been implicated in normal tactile function [40] and has been directly linked to hyperalgesia and allodynia in various pain animal models [22,31,41-43].

Strong evidence suggests that the T-type calcium channel, particularly CaV3.2, in DRGs is involved in the initiation and maintenance of chronic neuropathic pain. For instance, intrathecal treatment with the oligodeoxynucleotide antisense targeting CaV3.2 reduced normal nociception and neuropathic pain in rats [44]. These rats had decreased T-current density in corresponding lumbar DRG neurons, diminished expression of T-channel immunoreactivity in DRG tissue, and decreased CaV3.2 mRNA transcript expression in DRGs. Jagodic and colleagues have demonstrated similar events in nociceptive neurons (increased T-current density) following the chronic constriction injury (CCI) model of neuropathic pain in rats [45], while systemic injections of mibefradil and ethosuximide, both blockers of T-type calcium channels, reversed CCI-induced neuropathic pain [3]. Interestingly, Mibefradil does not cross the blood brain barrier in significant concentrations, and is thus devoid of CNS effects [4]. However, the blood nerve barrier surrounding the PNS is composed of fenestrated epineurialmicrovessels that do not possess tight junctions and would likely allow passage of substances (such as the peptides tested here) within the blood circulation into the contact with peripheral nerves and ganglia [46]. Pathirathna and co-workers [47] also reported that (3β,5α,17β)-17-hydroxyestrane-3-carbonitrile (ECN), a neuroactive steroid and T-channel blocker, alleviated neuropathic pain in rats with SNL. Conversely, up-regulation of CaV3.2/T-type channels has been show to contribute to the maintenance of neuropathic pain following spinal nerve injury [48]. T-type calcium channels have also been implicated in diabetic neuropathy models of neuropathic pain [22,31,49,50] with CaV3.2 knockout exhibiting attenuated acute pain responses [42]. In this report we cannot confirm that our peptide is acting specifically on the CaV3.2, however we report that TAT-CBD3 and TAT-CBD3A6K are effective in blocking T-type channels in sensory neurons and also effectively block AIDS therapy-induced painful peripheral neuropathy *in vivo*. These data reveal that TAT-CBD3A6K may be attenuating chronic neuropathic pain by decreasing sensory neuron neuronal excitability via blocking T-type calcium channels in the peripheral nervous system. Thus, these studies suggest that a major function of T-type calcium channels in DRG sensory neurons is to support nociceptive signals.

Though there is precedence for the role neuronal T-type channels play in the peripheral pain pathway, the association that R-type channels have with nociceptive processing in various pain conditions is less well-known [7,8]. R-type channels have been shown to be present in rat DRG neurons [10,11], where nociceptive small diameter DRG sensory neurons show higher levels of R-type channel mRNA than larger DRG neurons [51]. These primary afferents are thought to contribute to nociceptive processing in various nerve injury paradigms. For example, R-type channel knockout mice exhibit reduced responses to inflammatory pain stimuli [7,8], while nociceptive transmission in nerve injured rodents is inhibited by spinal administration of SNX-482 but not sham-operated control rats [7,8]. These results are supported by an earlier study by Murakami and colleagues [52] and together suggest that the influence that R-type currents exert in the nociceptive pathway is state-dependent and does not influence normal physiological pain. That administration of TAT-CBD3A6K effectively reduces both T- and R-type currents, reduces sensory neuron excitability, and attenuates neuropathic pain suggests that these calcium channels may both be important in pain transmission and neuropathic pain. Evidence linking block of T- and R-type calcium channels to analgesia can be surmised from the work of Dickenson and colleagues who reported that the CaV2.3 antagonist SNX-482 reduces primary afferent-mediated dorsal horn neuronal responses in a rat model of chronic neuropathic pain (spinal nerve ligation) [8] and Li and colleagues who reported that intrathecal administration of the T-type channel blocker mibefradil suppresses thermal hyperalgesia and allodynia in the chronic compression of dorsal root ganglion (CCD) model of neuropathic pain [53].

As was previously reported, our parent peptide TAT-CBD3 decreased neuropeptide release from sensory neurons, reduced excitatory synaptic transmission in dorsal horn neurons and transiently reversed neuropathic pain due to an antiretroviral drug [15] via inhibition of N-type calcium channel, CaV2.2. Surprisingly, TAT-CBD3A6K did not alter depolarization-induced release of CGRP which is contrary to omega-conotoxin inhibitory release of CGRP by sensory neurons (via N-type channels) [13,54,55]. However, that capsaicin-evoked release was reduced by ~30% by TAT-CBD3A6K is consistent with the effects of TAT-CBD3 reported by us earlier [15]. Thus, we cannot rule out possible effects of TAT-CBD3A6K on CaV2.2 as Ca^2+^ currents via this channel were not tested here. Furthermore, the differential stimulus-dependent block of CGRP release observed here may be dependent on (i) nerve growth factor (NGF)-sensitive sensory neurons as chronic exposure with NGF significantly and concentration-dependently serves to increase capsaicin-evoked CGRP release [56] or (ii) a requirement of a higher concentration for inhibition of CGRP release considering TAT-CBD3A6K seems to have a greater action on T- and R-type channels than TAT-CBD3 at the same concentration (Figure 7C). Importantly, our data suggest that TAT-CBD3A6K peptide has multiple targets of action – within the calcium channel family – all of which may contribute to anti-nociception. Although not tested here, it is possible that TAT-CBD3A6K’s mechanism of action may involve binding to T- and R-type channels in their first intracellular loops or distal parts of the carboxyl-termini – regions which we have previously demonstrated to be the sites of binding of TAT-CBD3 [15]. Sequence alignments reveal relatively high homology between the initial segment of the first intracellular loop of N- compared to R-, T-type channels but little to no homology in the remaining 60% of the first intracellular loop or the carboxyl termini of these channels (data not shown). We also show that the wild type TAT-CBD3 peptide reduces currents via T- but not R-type calcium channels. Importantly, N-type calcium channels are present in DRG neurons with T-type calcium channels suggesting that these peptides may interact with several subtypes of calcium channels in the same neurons [57]. That the block develops over minutes suggests that the peptides are not direct channel blockers and is consistent with our earlier findings that TAT-CBD3 works, at least in part, by interfering with anterograde trafficking of CaV2.2 [15]. This time frame of ~10 min is entirely compatible with an effect on trafficking; for example, acute activation of phosphatidylinositol 3-kinase by insulin-like growth factor 1 in DRG neurons leads to rapid translocation of CaV2.2 to the plasma membrane [58]. Moreover, our observation that the TAT-CBD3 containing scaffold peptides inhibit N, T-, and R-type currents suggests these peptides may disrupt a generalized mechanism of calcium channel trafficking.

It is possible that other ion channels that control neuronal excitability, such as Na^+^ or K^+^, could be modulated by our peptide. However, we have previously reported that our parent peptide, TAT-CBD3, does not alter Na^+^ channels [15] nor does full-length CRMP-2 [13], likely ruling out Na^+^ channel inhibition as a cause of the decreased neuronal excitability observed here. Ca^2+^ influx via HVACC is also coupled to big conductance Ca^2+^-dependent K^+^ (BK_Ca_) channels [59] which contribute to the modulation of both excitability and AP duration in rat DRG sensory neurons [60,61]. It remains to be tested if BK_Ca_ channels are affected by our peptide given its effect of reducing calcium via R- and N-type HVACC.

## Conclusions

These studies revealed that systemic administration of a structurally stable TAT-CBD3A6K peptide reverses d4T-induced nociceptive behavior and reduces currents via T-and R-type calcium channels implicating an interaction with CRMP-2 as being an important contributor of hyperexcitability of sensory neurons. The discovery of TAT-CBD3A6K offers a new approach for modulating individual Ca^2+^ channels and treatment of neuropathic pain. The present demonstration of the ability of TAT-CBD3A6K extends the potential therapeutic utility of T- and R-type calcium channel blockers in neuropathic pain states.

## Methods

### Animals

Pathogen-free, adult female Sprague–Dawley rats (150–200 g; Harlan Laboratories, Madison, WI) were housed in temperature (23 ± 3°C) and light (12-hlight: 12-h dark cycle; lights on at 07:00 h) controlled rooms with standard rodent chow and water available ad libitum. Experiments were performed during the light cycle. These experiments were approved by the Institutional Animal Care and Use Committee of Indiana University/Purdue University in Indianapolis. All procedures were conducted in accordance with the Guide for Care and Use of Laboratory Animals published by the National Institutes of Health and the ethical guidelines of the International Association for the Study of Pain. All animals were randomly assigned to either treatment or control groups.

### Peptides

TAT-CBD3 (YGRKKRRQRRRARSRLAELRGVPRGL; the transduction domain of the HIV TAT protein is indicated in the underlined text), TAT-CBD3A6K (YGRKKRRQRRRARSRL*K*ELRGVPRGL; italicized residue denotes a lysine instead of an alanine at that position) and TAT-CBD3 reverse (YGRKKRRQRRRLGRPVGRLEALRSRA; a reversed version of the CBD3 sequence with no homology to any known sequence) were synthesized by GenScript USA Inc. (Piscataway, NJ) and verified by mass spectroscopy (Department of Chemistry, IUSM) prior to use. Peptides were dissolved in saline prior to use.

### Purification/Enrichment of Ca^2+^channels from synaptosomes

To prepare a rich source of Ca^2+^ channels for the far-Western analyses (see below), synaptosomes from postnatal day 1 (PN1) rat brains were solubilized with digitonin and enriched by chromatography on WGA-Sepharose as described previously [62-64]. Briefly, 25 PN1 rat brains were homogenized in 180 ml of 320 mM sucrose with a glass-Teflon homogenizer. After a short centrifugation (5000 rpm, 2 min), the supernatant (SN) was centrifuged (42,000 rpm, 60 min). The membranes were solubilized with 1.2% digitonin, 80 mM sodium phosphate buffer, pH 7.4 for 20 min. Unsolubilized material was removed by the centrifugation as before, and the supernatant (S3) was poured over a 40 ml WGA-Sepharose column (50 ml/h). After incubation for 1 hr at 4°C, the column was washed with 10 column volumes of 0.1% digitonin, 75 mMNaCl, 50 mM sodium phosphate, 10 mMTris–HCl (pH 7.4) at a flow rate of 50 ml/hr. The glycoproteins bound to the WGA-Sepharose column were eluted with 100 mM N-acetyl-D-glucosamine (Sigma, St. Louis, MO) in the same buffer at a flow rate of 50 ml/hr. Three milliliter fractions were collected and the protein concentration of each fraction was determined by BCA protein assay kit (Thermo Fisher Scientific, Shelbyville, IN).

To further enrich for Ca^2+^ channels, WGA-column fractions were incubated for 4 hr on ice with 200 μl of heparin-agarose [65]. The resin was washed four times with 0.2% CHAPS, 10 mMTris–HCl, pH 7.4, and once with 10 mMTris–HCl, pH 7.4. Ca^2+^ channels were gently extracted for 30 min at 50°C with 100 μl of 5% SDS, 20 mMdithiothreitol, 125 mMTris–HCl, pH 6.8, 10% sucrose, 20 mM EDTA.

### Peptide spots arrays and Far Westerns

Peptide arrays harboring amino acid changes at every single position in the 15 amino acid length of CBD3 were constructed using the SPOTS-synthesis method [66-69]. Standard 9-fluorenylmethoxy carbonyl (Fmoc) chemistry wasused to synthesis the peptides on cellulose membranes prederivatized with a polyethylene glycerol spacer (Intavis AG, Cologne, Germany). Fmoc protected and activated amino acids (Intavis) were spotted in arrays on 150 mm by 100 mm membranes using the IntavisMultiPep robot. Membranes were probed in a far-Western manner with an antibody against CaV2.2 (CalbiochemInc, La Jolla, CA)[12]. Briefly, peptides were immobilized on a nitrocellulose membrane which was then soaked in CAPS buffer (10 mM CAPS pH 11.0 and 20% methanol) for 30 min, washed once with TBST, and then blocked for 1 h at room temperature with gentle shaking in TBST containing 5% non-fat milk and finally incubated with a purified synaptosome fraction enriched in Ca^2+^ channels for 1 h at room temperature with gentle shaking. Next, the membrane was incubated in primary antibody for CaV2.2 for 2 h at room temperature with gentle shaking, followed by washing with TBST. Finally, the membrane was incubated in secondary antibody (horseradish peroxidase-conjugated goat anti-rabbit; 1:10,000) for 45 min, washed for 30 min in TBST and developed using enhanced chemiluminescence.

### Molecular dynamics (MD) simulations of CBD3 wildtype and mutant peptides

The structure of the wild-type and A6K mutant peptides were generated using PyMOL (Version 1.5.0.1 Schrödinger, LLC). The coordinates were loaded into Maestro (version 9.2, Schrödinger, LLC, New York, NY). The program was used to protonate the peptides and assign atomic charges. Energy minimization was carried out using the Impact module of the Schrodinger package. The resulting structures were immersed into a TIP3P [70] water molecule such that no atom in the complex was within 14 Å from any side of the box. The peptides were neutralized with Na^+^ or Cl^-^counterions using the Leap program from the package [71]. An annealing process equilibrated the solvated structures before production runs were carried out using the pmemd module in AMBER. MD snapshots were saved every 1 ps, yielding 10,000 structures per trajectory. By assigning different initial velocities, 10 independent trajectories of 10 ns in length were collected for each of the peptides. The first 2 ns of trajectories were cut off for equilibrium using ptraj program in AMBER. 1,000 snapshots were selected from every 8n s trajectory. The root-mean-square deviation (RMSD) for backbone atoms was determined using 1,000 snapshots collected from each of the 10 trajectories per peptide.

### d4T model of peripheral neuropathy

Hyperalgesia and allodynia were established by a single injection (50 mg/kg) of the antiretroviral drug 2′,3′-didehydro-3’-deoxythymidine (d4T, Sigma) given i.p. A single administration of d4T produced a significant bilateral decrease in paw withdrawal threshold to von Frey hair stimulation from post-injection day (PID) 3 through the last day of testing at PID21 [72].

The von Frey test was performed on the area of the hind paws as previously described [73-76]. Briefly, the rat was placed on a metal mesh floor and covered with a transparent plastic dome wherein the animal rested quietly after an initial few minutes of exploration. Animals were habituated to this testing apparatus for 15 minutes a day, two days prior to pre-injection behavioral testing. Following acclimation, each filament was applied to 6 positions spaced across the glabrous side of the hind paw; two distinct locations for the distribution of each nerve branch (saphenous, tibial and sural). Mechanical stimuli were applied with 7 filaments, each differing in the bending force delivered (10, 20, 40, 60, 80, 100, and 120 mN), but each fitted a flat tip and a fixed diameter of 0.2 mm. The force equivalence of mN to grams is: 100 mN = 10.2 grams. The filaments were tested in order of ascending force, with each filament delivered for 1 second in sequence from the 1st to the 6th spot alternately from one paw to the other. The inter-stimulus interval was 10–15 seconds. A cutoff value of 120 mN was used; animals that did not respond at 120 mN were assigned that value.

Measurements were taken on 3 successive days before rats were subjected to either a TAT-CBD3 (30 mg/kg and 10 mg/kg) TAT-CBD3A6K (10 mg/kg) and TAT-CBD3 reverse (10 mg/kg). Stimuli were applied randomly to left and right hind paws to determine the stimulus intensity threshold stiffness required to elicit a paw withdrawal response. The incidence of foot withdrawal was expressed as a percentage of six applications of each filament as a function of force. A Hill equation was fitted to the function (Origin version 6.0, Microcal Software) relating the percentage of indentations eliciting a withdrawal to the force of indentation. From this equation, the threshold force was obtained and defined as the force corresponding to a 50% withdrawal rate. A threshold that exhibits at least a −20 mN difference from the baseline threshold of testing in a given animal is representative of neuropathic pain [76].

Threshold values were statistically analyzed for each foot separately and the significance of differences between the average of at least two pre-injection tests and the mean obtained for each post-injection test. In all tests, baseline data were obtained for the d4T-treated and sham-treated groups before drug or vehicle administration. Within each treatment group, post-administration means were compared with the baseline values by repeated measures analyses of variance (RMANOVA) followed by post hoc pairwise comparisons (Student-Newman-Keuls Method). A probability level of p <0.05 indicates significance.

### Preparation of acutely dissociated dorsal root ganglion neurons

Lumbar 1–6 (L1-L6) DRGs were acutely dissociated using methods described previously [77]. Briefly, L1-L6 dorsal root ganglia (DRGs) were removed from naïve Sprague–Dawley rats. The DRGs were treated with collagenase A and collagenase D in HBSS for 20 minutes (1 mg/ml; Roche Applied Science, Indianapolis, IN), followed by treatment with papain (30 units/ml, Worthington Biochemical, Lakewood, NJ) in HBSS containing 0.5 mM EDTA and cysteine for ~ 20 min at 35°C. The cells were then dissociated via mechanical disruption in culture media containing 1 mg/ml bovine serum albumin and trypsin inhibitor (1 mg/ml, Sigma, St. Louis MO). The culture media was DMEM, Ham's F12 mixture, supplemented with 10% fetal bovine serum, penicillin and streptomycin (100 μg/ml and 100 U/ml), N2 (Life Technologies) and 20 nM NGF (Sigma-Aldrich, St. Louis, MO). The cells were then plated on coverslips coated with poly-L lysine and laminin (1 mg/ml) and incubated for 2–3 hours before the wells were flooded with additional media. The cells were then allowed to sit undisturbed for 4–18 hours to adhere at 37°C (with 5% CO_2_) prior to electrophysiology.

### Current clamp electrophysiology

Electrophysiology experiments were performed using a modified method described previously [13,77]. Sharp-electrode intracellular recordings were obtained 4–18 h after dissociation. Coverslips were transferred to a recording chamber that was mounted on the stage of an inverted microscope (Nikon Eclipse Ti, Nikon Instruments Inc., Melville, NY). The chamber was perfused with a bath solution containing (mM): NaCl 120, KCl 3, CaCl_2_ 1, MgCl_2_ 1, Hepes 10, Glucose 10, adjusted to pH 7.4 and osmolarity 300 mosM. The recordings were obtained at room temperature. Intracellular recording electrodes were fabricated from borosilicate glass (World Precision Instruments, Sarasota, FL) and pulled on a Flaming/Brown micropipette puller (P-98, Sutter Instruments, Novato, CA). Electrodes were filled with 1.0 M KCl (impedance: 40–80 MΩ) and positioned by a micromanipulator (Newport Corporation, Irvine, CA). Negative 0.1 nA current injection was used to bridge-balance the electrode resistance. Prior to electrode impalement, the size of the soma to be recorded was classified according to its diameter as small (≤30 μm), medium (31–45 μm) and large (≥45 μm). Only small-to-medium size neurons were used in these experiments as these neurons are considered to have nociceptive properties and express T-currents [22,38,78]. Electrophysiological recordings were performed with continuous current-clamp in bridge mode using an AxoClamp-2B amplifier, stored digitally via Digidata 1322A interface, and analyzed offline with pClamp 9 software (Molecular Devices, Inc., Downingtown, PA). A neuron was accepted for study only when it exhibited a resting membrane potential (RMP) more negative than −45 mV. Action potentials were evoked by injecting current steps of 1 s duration through the intracellular recording electrode from 0.1 nA in increments of 0.1 nA until evoking 4–6 action potentials per current pulse, or reaching 0.4 nA. Baseline neuronal excitability was measured by injecting 1-s current pulses into the soma every 30 s. Following 3 control current injections, peptides were applied to the coverslip (final concentration of 10 μM) and current injections continued every 30 s. Neuronal excitability was measured as number of action potentials elicited per current pulse before and after addition of peptides.

### Whole cell voltage-clamp electrophysiology

Recordings were obtained from acutely dissociated DRG neurons as described previously [13]. To isolate calcium currents, Na^+^ and K^+^ currents were blocked with 1 μMtetrodotoxin (TTX; Alomone Laboratories), and 30 mMtetraethylammonium chloride (TEA-Cl; Sigma). To isolate T-type calcium currents cells were held at −90 mV and bathed in recording solutions containing 5 μM Nifedipine (Sigma), 500 nM ω-Conotoxin GVIA (Alomone Laboratories), and 200 nM ω-Agatoxin IVA (Alomone Laboratories). Extracellular recording solution (at ~315 mOsm) consisted of (in mM): 110 N-methyl-d-glucamine (NMDG), 10 BaCl_2_, 30 TEA-Cl, 10 HEPES, 10 glucose, 0.001 TTX, 0.005 nifedipine, 0.0005 ω-Conotoxin GVIA, and 0.0002 ω-Agatoxin IVA. The intracellular recording solution (at ~305 mOsm) consisted of (in mM): contained 150 CsCl_2_, 10 HEPES, 5 Mg-ATP, 5 BAPTA, pH at 7.2 with KOH. Fire polished recording pipettes, 2–5 MΩ resistances were used for all recordings. Whole cell recordings were obtained with a HEKA EPC-10 USB (HEKA Instruments Inc.); data were acquired with Patchmaster (HEKA) and analyzed with Fitmaster (HEKA). Capacitive artifacts were fully compensated and series resistance was compensated by ~70%. Recordings made from cells with greater than a 5 mV shift in series resistance compensation error were excluded from analysis. All experiments were performed at room temperature (~23°C). The currents were filtered at 5 kHz and sampled at 2 kHz using Patchmaster (HEKA).

### Whole cell voltage protocols and data analysis

In order to isolate T-type calcium currents cells were held at −90 mV. After obtaining whole cell configuration, cells were allowed to equilibrate for 2 minutes before data acquisition began. During data acquisition several protocols were executed including ramp depolarizations from −60 mV to +20 mV for 2 sec and step depolarizations from −60 mV to +50 mV in 5 mV increments. Leak currents were subtracted online using a –P/5 procedure. The activation of Ca^2+^ channels from DRGs was well described by the Boltzmann relation: G/Gmax = 1/{1 + exp[(V-V50%)/kn]}, where G is peak conductance, Gmax is fitted maximal G, V50% is half-activation voltage, and kn is the slope factor. Data were acquired using a HEKA EPC-10 amplifier (HEKA) and analyzed using Fitmaster (HEKA) and Origin 8.0 software (Microcal, Northampton, MA).

### Stimulated iCGRP release from rat DRGs in culture

Measurement of stimulus-evoked release and content of immunoreactive CGRP (iCGRP) from isolated sensory neurons was performed as published [13,15]. After 12–14 days in culture, the basal or resting release of iCGRP was measured from aliquots of media removed from cells incubated for 10 minutes in non-depolarizing HEPES buffer consisting of (in mM): 25 HEPES, 135 NaCl, 3.5 KCl, 2.5 CaCl_2_, 1 MgCl_2_, 3.3 dextrose, and 0.1% (w/v) bovine serum albumin, pH 7.4, and maintained at 37°C. The buffer was removed and the cells were re-exposed to the same HEPES buffer in the presence of peptide or vehicle control and then incubated in either a depolarizing HEPES buffer (50 mM KCl) or a capsaicin-containing HEPES buffer (30 nM Cap) for 10 minutes, following which media was removed and aliquotted. The cells were finally incubated again with non-depolarizing HEPES buffer with 3.5 mM KCl to re-establish resting release levels, and the buffer was removed and aliquotted. The amount of iCGRP released was measured in aliquots of the incubation samples by a radioimmunoassay (RIA). The minimum amount of iCGRP detected by the RIA is 5 fmol with a 95% confidence interval68. The remaining peptide content in each well was determined by exposing the cells to 2 N acetic acid for 10 minutes. Aliquots of the acid solution were diluted with HEPES buffer and similarly assay for iCGRP. This value was added to the amount of iCGRP released in the previous incubations to yield the total iCGRP content per well. The release of iCGRP during the 10 min incubation periods is expressed as percent of the total content. DRGs were either exposed to TAT-CBD3A6K peptides (10 μM) throughout the first basal wash and throughout the high K^+^ exposures. A minimum of three different preparations were used for each condition.

## Abbreviations

AP: Action potential; BK_Ca_: Big conductance Ca^2+^-dependent K^+^ channels; CRMP-2: Collapsin response mediator protein; CaV2.2: N-type voltage-activated Ca^2+^ channel; CaV3.x: T-type voltage-activated Ca^2+^ channel; CaV2.3: R-type voltage-activated Ca^2+^ channel; CBD: CaV binding domain on CRMP-2; Cap: Capsaicin; CBD3A6K: CBD3 with alanine to lysine mutation; CCI: Chronic constriction injury; CGRP: Calcitonin gene related peptide; d4T: 2^′^,3^′^-didehydro-3’-deoxythymidine; DIV: Days *in vitro*; DRG: Dorsal root ganglion; HVACC: High voltage activated calcium channels; LVACC: Low voltage activated calcium channels; ω-Aga: ω-Agatoxin; ω-CTX: Omega-conotoxin GVIA; MD: Molecular dynamics; Nif: Nifedipine; RMSD: Root mean square deviation; SNL: Spinal nerve ligation.

## Competing interests

The authors declare that they have no competing interests.

## Authors contributions

ADP performed whole-cell electrophysiology. MRD performed excitability experiments and wrote manuscript. MK performed peptide simulations. BW and SOM performed molecular dynamics simulations and wrote that section. MSR performed the behavior experiments. RW performed the iCGRP release experiments. MRV reviewed/edited the manuscript. FAW reviewed/edited the manuscript. RK identified the peptide, conceived the study, designed and supervised the overall project and wrote the manuscript. All authors read and approved the final manuscript.

## Supplementary Material

Additional file 1 **Figure S1.** Movie illustrating molecular dynamics simulations of the wild type CBD3 and the mutant CBD3A6K peptides. Click here for file
